# Stability of human gut microbiome: Comparison of ecological modelling and observational approaches

**DOI:** 10.1016/j.csbj.2023.08.030

**Published:** 2023-08-29

**Authors:** Anastasia Revel-Muroz, Mikhail Akulinin, Polina Shilova, Alexander Tyakht, Natalia Klimenko

**Affiliations:** a Center for Precision Genome Editing and Genetic Technologies for Biomedicine, Institute of Gene Biology, Russian Academy of Sciences, Moscow, Russia; b Department of Biological and Medical Physics, Moscow Institute of Physics and Technology, Institutskiy per. 9, Dolgoprudny, Moscow Region, Russia; c Department of Biology, Moscow State University, 1–12 Leninskie Gory, Moscow, Russia; d Atlas Biomed Group - Knomx LLC, Interchange House, Office 1.58, 81–85 Station Road, Croydon CR0 2AJ, United Kingdom

**Keywords:** Human gut microbiome, Stability, Responders, Microbiome dynamics, Dietary interventions, Time series, Meta-analysis

## Abstract

The gut microbiome plays a pivotal role in the human body, and perturbations in its composition have been linked to various disorders. Stability is an essential property of a healthy human gut microbiome, which allows it to maintain its functional richness under the external influences. This property has been explored through two distinct methodologies - mathematical modelling based on ecological principles and statistical analysis drawn from observations in interventional studies. Here we conducted a meta-analysis aimed to compare the two approaches utilising the data from 9 interventional and time series studies encompassing 3512 gut microbiome profiles obtained via 16S rRNA gene sequencing. By employing the previously published compositional Lotka-Volterra method, we modelled the dynamics of the microbial community and evaluated ecological stability measures. These measures were compared to those based on observed microbiome changes. There was a substantial correlation between the outcomes of the two approaches. Particularly, local stability assessed within the ecological paradigm was positively correlated with observational stability measures accounting for the compositional nature of microbiome data. Additionally, we were able to reproduce the previously reported inverse relationship between the community's robustness to microorganism loss and local stability, attributed to the distinct impacts of coefficient characterising the network decomposition on these two stability assessments. Our findings demonstrate harmonisation between the ecological and observational approaches to microbiome analysis, advancing the understanding of healthy gut microbiome concept. This paves the way to develop efficient microbiome-targeting interventions for disease prevention and treatment.

## Graphical Abstract

**Figure:**
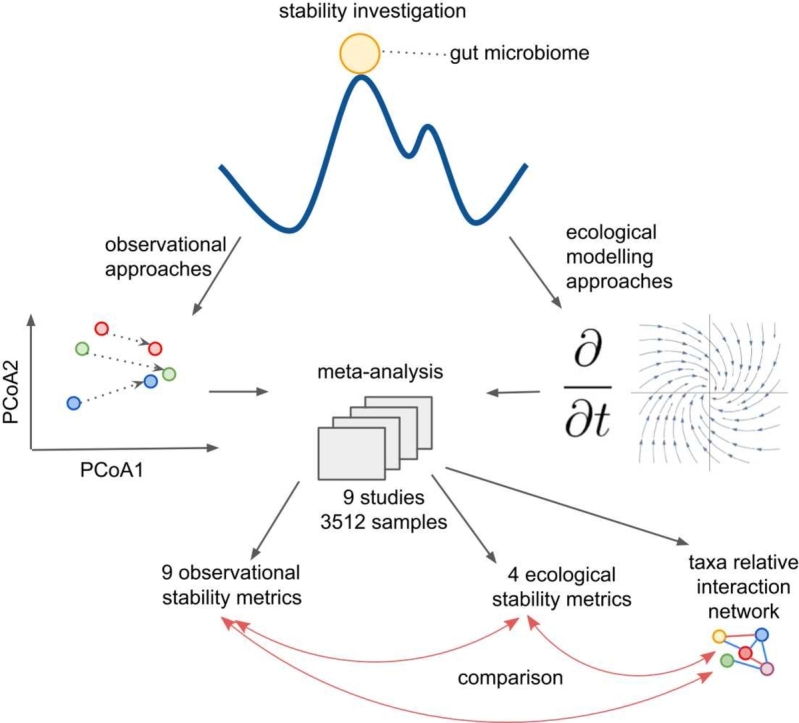

## 1 Introduction

The stability of the intestinal microbiome is closely related to human health as the microbiome plays a crucial role in various physiological processes [1]. Currently, there are two distinct approaches to the stability analysis of the gut microbiome. Firstly, it is a special case of complex living systems stability problem, which has been explored extensively in the field of microbial ecology. These studies have revealed fundamental principles governing the dynamics of complex microbial systems through mathematical modelling. The growing body of data on the dynamics of gut microbiome composition has created an opportunity to parameterize mathematical models and apply this analytical framework to study intestinal community stability. On the other hand, in parallel, observational approaches for studying gut microbiota stability have started to develop recently. The accumulated microbiome data includes a vast number of interventional studies that have observed significant inter-individual variability in microbiome response to perturbations. These observations have prompted researchers to investigate the underlying mechanisms of intestinal community stability through statistical analysis. In the following paragraphs, we provide a summary of the current state of affairs regarding stability in each of these two fields.

In microbial ecology, various approaches are utilised to assess stability. One widely employed method is local (asymptotic) stability analysis of a steady state [2], [3], [4], [5]. This approach characterises the system’s behaviour near the equilibrium under small perturbations. Mathematically, it involves calculating the eigenvalues of the Jacobian matrix of the microbial dynamic system. If the real parts of all eigenvalues are negative, the system will return to equilibrium after a small perturbation and is considered locally stable. The concepts of D-stability and global stability are closely related to local stability. D-stability implies the local stability of all steady states, while global stability implies the stability of all steady states regardless of the magnitude of external influences [6]. One of the pioneering studies on the local stability of complex communities is the fundamental work of May [5], where he introduced the concept of a trade-off between complexity and stability. According to it, the system remains stable up to a certain level of complexity, which is determined by the number of species, connectance and average interaction strength. In May's work [5] species interactions were sampled from random number distributions with fixed mean and square mean values. Subsequent studies have advanced the formalisation of community dynamics by considering different proportions of interaction types, including predator-prey, mutualistic, or competitive relationships [2], [4], [7], [8]. Essential advancements in stability analysis include incorporation of interactions with saturation [8] and system feasibility consideration [6], where system feasibility implies that all species have non-negative abundances. Interestingly, several studies that have taken these properties into account have shown that local stability is guaranteed for a wide range of interaction types and strengths [3], [6], [8]. This highlights the significance of combining local stability analysis with other stability estimation approaches.

One of them is external stability, which refers to the community's ability to resist the invasion of new species [8], [9]. It is closely linked to another metric, the robustness of the community to species loss. Robustness can be quantified as the proportion of species that need to be lost to trigger the secondary extinction of a certain percentage of species (typically 50%) [10], [11]. External stability and robustness are often evaluated through *in silico* experiments [11]. Structural stability is another important concept in stability analysis, referring to a system's ability to maintain its dynamic behaviour under smooth changes in parameter values [3], [6], [9], [12]. The size of the parameter space where the system remains feasible and locally stable indicates the range of conditions under which the stable coexistence of the community is possible. When the feasibility regions of two or more steady states overlap, it creates conditions for multistability and regime shifts within the system [9].

Several studies have effectively parameterized mathematical models describing microbiome dynamics using gut microbiome profiles, mainly derived from gnotobiotic mice, obtained through next-generation sequencing methods [7], [13], [14], [15], [16], [17], [18], [19], [20], [21], [22]. These studies have significantly advanced our understanding of intestinal communities, particularly in relation to stability [7], [13], [14], [19], [23], [24]. The following are some notable discoveries:

1) The application of the concept of local stability to gut microbiomes revealed the existence of multiple stable states and regime shifts triggered by external perturbations [13].
2) The integration of local stability and external stability concepts, specifically in relation to the invasion of *Clostridium difficile*, led to the identification of a minimal subcommunity capable of resisting pathogen invasion [23].
3) Healthy microbiomes demonstrated a higher likelihood of local stability compared to dysbiotic ones. This finding was further supported by an alternative measure of stability, conceptually similar to robustness, involving *in silico* modelling of system behaviour under perturbation with antibiotics [14].
4) Competitive cycles within interaction networks were identified as potential drivers of instability in dysbiotic microbiomes [14].

It is important to note that parameterizing the dynamics of the gut microbiome is a difficult task because of the community's inherent complexity, the sparsity and compositionality of the microbiome data, and the limited availability of large longitudinal datasets [25], [26], [27]. In several studies, the analysis of gut microbiome stability within the framework of theoretical ecology has been conducted without explicitly parameterizing dynamic systems [26], [28], [29], [30]. These studies have contributed to the analytical foundation of enterotypes, demonstrating that multistability arises from the heterogeneity in the microbial interaction network [30] and confirming the existence of a complexity-stability trade-off in human-associated microbiomes [28].

In contrast, studies primarily devoted to the analysis of real microbiome data have defined stability in a fundamentally different manner. In this context, stability is estimated as the opposite value of the observed changes, which in turn are typically measured as weighted or unweighted beta diversity between relative microbiome profiles at two time points [31], [32], [33], [34], [35], [36]. The values of the observed changes themselves are also investigated and referred to as "temporal variability" or "response" [37], [38]. Through longitudinal analyses using beta diversity, it has been demonstrated that microbiome samples from the same individual, even when separated by time intervals of years, tend to be more similar compared to samples obtained from different individuals simultaneously [33], [39]. Interventional and time series studies have revealed that the gut microbial community is capable of partially or completely restoring its original state after external perturbations [39], [40]. These observations have led to the concept of gut microbiome stability based on observational approaches. Further investigations have revealed that under the same environmental conditions, the microbiomes of different individuals show varying degrees of temporal variability or response to specific interventions [34], [41], [42]. This raises the question of which factors determine the microbiome response or stability, including host-associated factors, external factors, and intrinsic ecosystem factors. In particular, intrinsic ecosystem factors are of special interest when studying gut microbiome communities as ecological systems. Among these factors, functional redundancy has been identified as a key determinant of microbiome stability [1], [34], [38], [43], [44]. A high level of functional redundancy is a hallmark of a healthy stable microbiome. However, there have also been observations of unhealthy stable states, such as those associated with inflammatory bowel diseases or recurrent *Clostridium difficile* infection [1], [43], [44]. While the transition of the community from these unhealthy stable states to healthier ones is challenging, the mechanisms underlying the stability in such cases are still being explored [1].

In this research paper, we have made one of the initial attempts to draw parallels between the two aforementioned approaches for assessing gut microbiome stability. Through a meta-analysis of 3512 gut microbiome profiles from time series and interventional studies, we have determined which methods from these two approaches yield similar results. Additionally, we have investigated the parameters of the microbial relative interaction network that are associated with stability in each case.

## 2 Results

### 2.1 Collected data

**Fig. 1:**
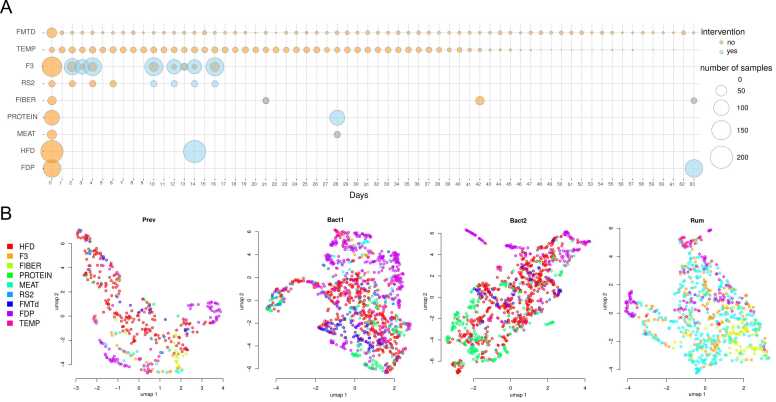
Summary of the analysed microbiome data. A - Distribution of samples included in meta-analysis by time points for 9 studies. The colour denotes whether the sample was collected after the intervention or not. B - Distribution of samples included in the meta-analysis by enterotypes visualised using the UMAP algorithm [48] (Prev: N = 411, Bact1: N = 1212, Bact2: N = 1083, Rum: N = 806).

For the purposes of gut community stability analysis, we collected microbiome profiles of adult individuals obtained through 16S rRNA gene sequencing. The data was sourced from 9 interventional and time series studies, resulting in a total of N = 3512 samples from S = 755 subjects (Table 1, Supplementary Table 1). We limited the duration of the time series to 2 months (63 days) to exclude long periods during which uncontrolled external influences were more likely to occur. The number of time points per individual varied from 2 to 56, with time intervals between adjacent points ranging from 1 to 63 days (Fig. 1). The studies used different experimental designs, including time series without interventions, case-control studies, and crossover interventions. Among the collected data, 659 subjects were from 6 studies with no external influences, which included time series studies (3 studies) and placebo groups in interventional studies (3 studies). Also, there were 10 different dietary interventions, which can be categorised into the following groups:

1. overall dietary pattern changes, such as high-fibre diets or substitution of meat products with alternative protein sources (3 studies, 317 subjects);
2. intake of prebiotics and specific high-fibre products (3 studies, 188 subjects);
3. intake of probiotic-fortified products (1 study, 129 subjects).

All participants were generally healthy. The studies were conducted in four countries: Belgium, the USA, the UK, and Russia. The participant demographics included 45% of men and 55% of women, with the age range from 17 to 65 years (statistics were collected using available data, some information was not available for PROTEIN, HFD, FMTD and F3 studies). Despite noticeable differences in taxonomic composition among the studies (PERMANOVA p = 0.001, Aitchison distance), the samples did not exhibit clear clustering by study (Supplementary Figure 1). For further analysis, the subjects were classified into four enterotypes based on the most frequent enterotype observed across all their samples. The detected enterotypes were similar to those described earlier in terms of the most abundant taxa profiles (Supplementary Figure 2) [45], [46], [47]. They included:

1. Bacteroides 1 enterotype (Bact1) where *Bacteroides* prevailed (number of subjects S=197, number of samples N = 1212);
2. Bacteroides 2 enterotype (Bact2) where *Bacteroides* prevailed and the lowest community diversity was observed (S=145, N = 1083);
3. Prevotella enterotype (Prev) where *Prevotella* prevailed (S=88, N = 411);
4. Ruminococcus enterotype (Rum) where *Faecalibacterium* prevailed and the highest community diversity was observed (S=324, N = 806).

Each resulting enterotype included subjects from different studies, and all studies included subjects from more than one enterotype (Fig. 1, Supplementary Figure 3).

### 2.2 Enterotype-specific and unstratified models of gut microbiome dynamics

In order to calculate ecological microbiome stability measures, such as local stability and robustness, it is necessary to create a model of community dynamics. We employed the compositional Lotka-Volterra (cLV) method, which has been recently developed for modelling microbiome dynamics using relative abundances [15] and demonstrated good performance [14], [15]. This approach effectively addresses the issue of compositionality by modelling the dynamics of taxa abundances in relation to the abundance of a selected taxon hereinafter referred to as denominator taxon. The model parameters include relative growth rates, a relative interaction matrix, and relative influences of external effects (see Materials and methods). The cLV algorithm automatically selects a denominator taxon with potentially minimal influences from other taxa, aiming to bring the relative interaction matrix closer to what would be obtained by applying the generalised Lotka-Volterra model to absolute taxa abundances [15].

We used the collected data to infer the parameters of the cLV model. During the inference process, we made several assumptions, in addition to the inherent assumptions of the cLV model itself. These assumptions are briefly listed below and are further discussed in detail in the Discussion section:

1. Relative taxa growth rates, relative interactions between taxa and relative influences of external effects are universal across populations and subjects;
2. Taxa abundance dynamics can be modelled at the level of microbial genera;
3. Rare genera can be excluded from the community dynamics model;
4. The effects of external influences other than the reported interventions on gut microbiome dynamics were negligible;
5. The ecosystem dynamics parameters remained unchanged for 2 months.

It is important to acknowledge that these assumptions simplify the complexity of the real intestinal microbiome. However, the primary objective of our study is to obtain a general understanding of community stability rather than predict precise trajectories of taxa abundances. We hypothesise that even under these strong assumptions, it is possible to gain insights into important characteristics of system behaviour, such as stability.

**Fig. 2:**
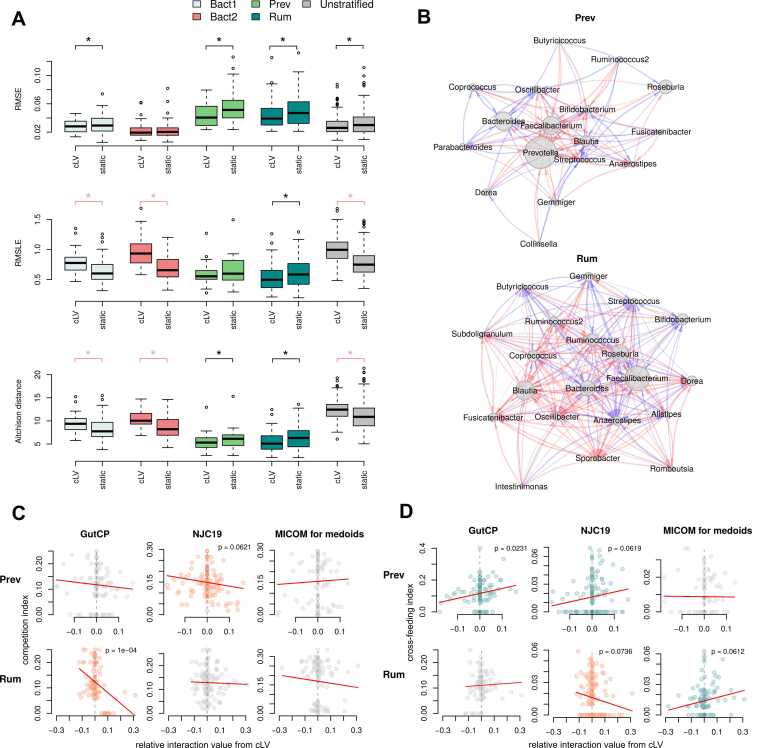
Performance of the microbial community dynamics models. A - Prediction quality accessed through leave-one-out cross-validation. Prediction quality of trained cLV was compared to static model considering no changes in microbiome composition. Black asterisks denote significant differences (Wilcoxon rank sum test, p < 0.05) between cLV and static model, where cLV showed better prediction quantity, and pink asterisks - lower quality. Number of subjects for testing: Bact1 50, Bact2 36, Prev 21, Rum 82 (summing up to 25% of the original 755 samples due to the regularisation step), and Unstratified 189. B - Relative interactions matrices for Rum and Prev enterotypes. Node size is proportional to taxon abundance in enterotype, red edges denote negative relative interactions and blue - positive. Edge width is proportional to interaction strength. C, D - Comparison of relative interaction coefficients revealed by cLV for Rum and Prev enterotypes with competition (C) and cross-feeding (D) indices calculated using external databases (see Materials and methods). Correlations that were considered significant (p < 0.1) are coloured in red (negative correlations) and blue (positive correlations).

We implemented model inference for the collected data using two approaches: unstratified (all data combined) and stratified by enterotypes. The number of model parameters and the corresponding data for each training option are provided in Supplementary Table 2. To assess the variability of model parameters, we performed subsampling within the same enterotype and compared it to the variability observed between enterotypes. We found that the variability of model parameters within the same enterotype was significantly lower than the variability between enterotypes (mean Pearson's R between relative interaction coefficients: 0.71 ± 0.08 vs. 0.51 ± 0.20). To assess the quality of the models, we conducted leave-one-out cross-validation (see Materials and methods). The results showed that the cLV model outperformed the static mode (no changes in abundances from the baseline sample) for both the unstratified training option and each of the Bact1, Rum, and Prev enterotypes (Fig. 2 A). This assessment was based on the root mean square error (RMSE), which was the optimisation metric during cLV model training. However, when using the root mean square log error (RMSLE) or compositional change (Aitchison distance) as evaluation metrics, performance improvement of the cLV model was observed only for the Rum and Prev enterotypes. One possible reason for this difference is that RMSLE and Aitchison distance calculations involve log transformation, which can amplify the relative contribution of low-abundant taxa. Due to this, all further analyses involving the obtained cLV models were conducted only for the subjects from Prev and Rum enterotypes (Fig. 2B).

Among the parameters derived from our models, the relative interaction matrices stand out as particularly significant for the subsequent stages of our investigation. To validate these parameters, we performed a comparative analysis of the relative interactions among genera using three distinct external sources. These sources encompassed:

1. previously published data on modelling microbial interactions via the GutCP algorithm applied to microbiome and metabolome data of school-aged children [49];
2. a literature-curated metabolic interaction network for the mammalian gut microbiome called NJC19 [50];
3. metabolic interaction networks, generated using the MICOM algorithm based on genome-scale metabolic models (GSMMs) for Prev and Rum enterotypes medoids (2 samples from our data) [51].

All three sources contain relations between metabolites and microbes, indicating the type of metabolic activity (import or export). Using this information, we calculated two indices for each pair of microbial genera in our data using the previously proposed concept [52]: the cross-feeding index, which considers the proportion of metabolites produced by one microbe from a pair and consumed by another, and the competition index, which considers the proportion of metabolites simultaneously consumed by both microbes from a pair (see Materials and methods). The aim was to assess whether the relative interactions obtained from the cLV model exhibited positive correlations with cross-feeding indices and negative correlations with competition indices, which would support the reliability of the obtained relative interactions. The Prev enterotype demonstrated consistent patterns of significant correlations with both the cross-feeding index (in GutCP and NJC19) and the competition index (in GutCP), supporting the findings related to relative interactions (Fig. 2 C, D). However, for the Rum enterotype, interactions were confirmed only by the competition index (in GutCP), while correlation with the cross-feeding index demonstrated both directions (in NJC19 and MICOM) (Fig. 2 C, D). Overall, the majority of the comparisons (9 out of 12) and significant correlations (5 out of 6) demonstrated consistent directions, providing additional support for the relative interactions revealed by our model.

### 2.3 Comparison of stability assessment using ecological modelling and observational approaches

The ecological stability metrics for individuals from the Rum and Prev enterotypes were estimated using the obtained cLV models. Firstly, we calculated genera abundances in steady states for each individual (see Materials and methods). Specifically, 325 baseline samples from the Rum enterotype converged to 51 unique steady states (out of them, one state attracted 64% and each of the rest - 1% on average), while 88 baseline samples from the Prev enterotype converged to 10 unique steady states (75% and 3%, respectively) (Supplementary Figure 4). All obtained microbiome compositions in steady states were feasible. The abundances of genera in the steady states were then used to calculate the real parts of eigenvalues of the Jacobian matrix of the dynamical systems, which provided an assessment of local stability. All steady states had eigenvalues with both positive and negative real parts, indicating that none of them were locally stable. The value of the rightmost eigenvalue's real part was used as a measure of steady state instability (R(λ_max_)). Additionally, the inverse distance from the baseline microbiome profile to the corresponding steady state was measured using Bray-Curtis (StP(1-BC)) and Aitchison dissimilarity metrics (StP(-Ait)). Furthermore, the robustness of each community was estimated using *in silico* simulations (Rob_0.5_). Robustness was defined as the percentage of initially removed taxa that led to a decrease of initial richness by 50% [11]. As a result, four stability estimates were obtained within the theoretical ecology framework (Supplementary Table 3).

As for the observational approaches to the stability estimation, the first step is to measure the inverse beta diversity between individuals' microbiomes at the first and second time points. Hereinafter, when we use the term "inverse", it should be understood in the sense of being multiplied by − 1 or subtracted from 1 (for the metrics ranging from 0 to 1). Various inverse beta diversity metrics were utilised, including Bray-Curtis (1-BC), Weighted Unifrac [53] (1-WUni), Euclidean distance after log transformation (1-Euc(log)), Aitchison [54] (-Ait), Jaccard (1-Jaccard), Raup–Crick metric based on Bray–Curtis dissimilarity (-RC_bray_) [55], and also Pearson's correlation coefficient (Cor). Additionally, the concept of response potential we recently proposed was employed [38]; it was calculated via each of the Bray-Curtis and Aitchison dissimilarity metrics, and stability was estimated as inverse response potential (P(1-BC) and P(-Ait)), respectively). As a result, a total of 9 observational stability estimates were obtained for each individual in the dataset (Supplementary Table 3). We calculated stability for baseline samples of all individuals in our data by utilising all 13 methods described above.

**Fig. 3:**
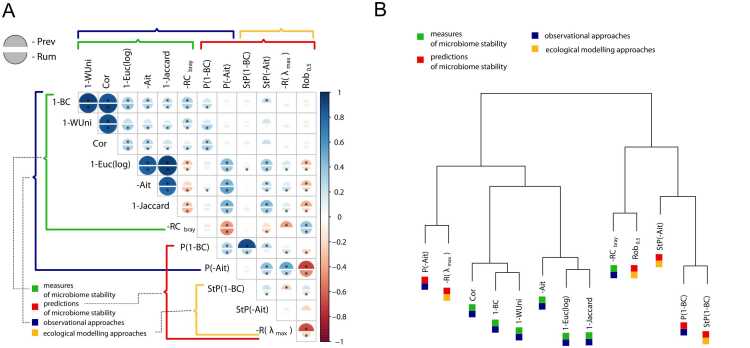
Links between model-based and observational stability estimates. A - Pearson’s correlation coefficients between observational and ecological modelling stability metrics. Stability metrics abbreviations are deciphered in the text. Colour and size of semicircles denote correlation coefficient, upper semicircle corresponds to Prev enterotype, and lower - to Rum. Asterisk denotes significant correlations (FDR<0.05). The number of samples for Prev/Rum enterotype is 88/325 respectively. B - Hierarchical clustering of the stability metrics based on the obtained correlations.

To compare all the stability estimates obtained, we calculated the Pearson correlation while adjusting for time interval, intervention, and alpha diversity (Fig. 3 A).

The inter-metrics correlation patterns were quite similar between individuals from Rum and Prev enterotypes. Among ecological modelling stability measures, we observed a negative correlation between local stability and robustness, which is consistent with a recently discovered trade-off between these two measures [11]. The key correlations between observational and ecological metrics included the following:

1. Local stability showed a positive correlation with observational stability measured by inverse Aitchison, Jaccard, and Euclidean distance after log transformation.
2. Local stability and inverse distances from steady states were positively correlated with inverse response potential, measured using each of the Bray-Curtis and Aitchison metrics.
3. Robustness exhibited a positive correlation with the measure of stability calculated using the RC_bray_ metric.

As a result of the hierarchical clustering, two distinct clusters of stability measures were observed (Fig. 3 B), which we named according to the ecological stability measures they included - the cluster of local stability and the cluster of robustness. We analysed whether the baseline microbiome signature for the samples from Rum and Prev enterotypes was associated with measures in each of the stability clusters (Supplementary Table 4). Significant correlations were observed for more than half of the measures in each cluster for the most abundant taxa in the enterotypes: *Prevotella* and *Bacteroides* in Prev and *Bacteroides*; *Faecalibacterium*, *Roseburia* and *Blautia* - in Rum. These associations were positive for the cluster of robustness and negative - for the cluster of local stability. The other highly reproducible associations between stability measures of each cluster included: for local stability - negative correlations with *Clostridium cluster XlVa* and *Dorea* and positive - with *Oscillibacter* and *Alistipes*; for robustness - negative correlation with *Collinsella*.

### 2.4 Relative interaction matrix characteristics associated with stability

Next, we compared the yielded stability estimates with the characteristics of the microbial interaction networks. We generated taxa relative interaction networks for each individual using the parameters of cLV models. We only considered taxa with non-zero baseline abundances in these networks. In cLV models, relative interactions are the differences between the absolute interactions and the absolute influence of each taxon on the denominator (*A*_*dj*_). In the original paper, introducing the cLV algorithm [15], it was shown that, under an appropriate selection of the denominator and moderate fluctuations of microbial load across samples, the relative interactions between microbes can partially mirror the absolute interactions, in particular, recover the interaction sign. In our dataset, we assume low variation in microbial load, since the external influences are dietary interventions that are not as destructive to the microbiome as antimicrobial therapy or pathogen invasion. During model construction, the denominator was chosen as the taxon with the lowest variance in log-abundance, which is expected to result in the lower values of *A*_*dj*_ [15]. Therefore, we expect that the signs of the relative interactions in our models would mostly match with the signs of the absolute interactions. This allows us to approximate the competition and mutualism strength (*S*_*c*_ and *S*_*m*_), as well as the absolute interaction strength (*S*) (see Materials and methods).

Regarding the ability of the relative interaction network to mirror the presence and absence of absolute interactions, one would expect to encounter certain distortions due to the subtraction of *A*_*dj*_. In the relative interaction network, the denominator node lacks incoming edges, and when taxon *j* has a non-zero influence on both taxon i and the denominator (*A*_*dj*_≠0 and *A*_*ij*_≠0), we will observe a single edge in the network instead of two. However, for all taxa but the denominator, the presence or absence of a certain interaction can be accurately determined, because the probability of *A*_*dj*_= -*A*_*ij*_ is relatively low. With these considerations in mind, we calculated the connectivity of the relative interaction network (*C*), the heterogeneity of node degrees (*H*), and the coefficient *b* characterising the network decomposition [11].

**Fig. 4:**
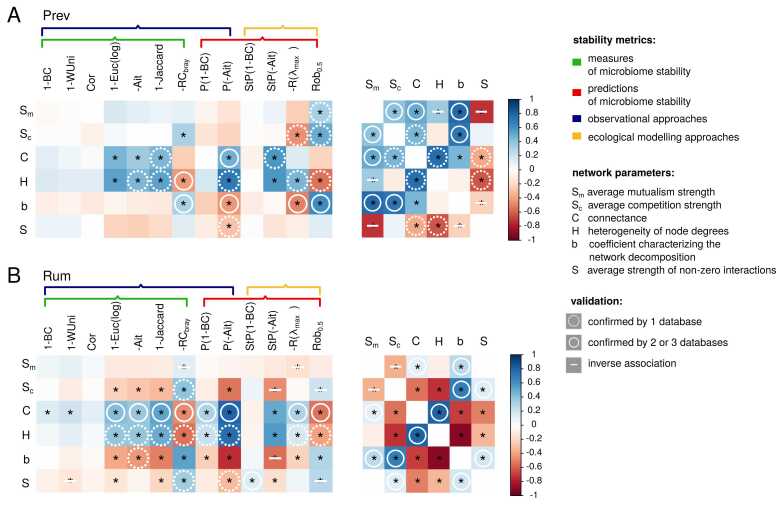
Correlations of relative interaction network characteristics - with stability estimates and within themselves. Stability metrics abbreviations are deciphered in the text. Colour denotes correlation coefficient. Asterisk denotes significant correlation (FDR<0.05). White circles and strikes denote associations which were also significant when external databases were used for network parameters estimation (GutCP, MICOM, NJC19) (FDR<0.05). The number of samples for Prev/Rum enterotype is 88/325 respectively.

To address potential distortions resulting from the use of relative interactions and improve the reliability of the estimated characteristics, we also calculated them using the 3 above-mentioned external databases (GutCP, MICOM, NJC19). We then compared all 6 characteristics of the relative interaction networks (*S*_*c*_*, S*_*m*_*, S, C, H*, and *b*) with the previously obtained stability estimates and validated the associations using the characteristics derived from the external databases (Fig. 4).

The associations of stability metrics with network properties varied considerably between the Rum and Prev enterotypes (Fig. 4). However, in both enterotypes, the networks with high heterogeneity of node degrees were characterised by increased connectance and simultaneously reduced strength of non-zero interactions. Networks with higher heterogeneity and connectance had reduced robustness and -RC_bray_ stability. At the same time, such networks manifested enhanced stability according to the local stability metric and a range of observational metrics (including those based on Aitchison, Jaccard, and Euc(log) distances), as well as metrics based on response potential (P(-Ait)). Similar to the previously published work [11] the trade-off between robustness and local stability was affected by the opposite dependence of these metrics on the coefficient b characterising network decomposition. As for the interaction types, a higher competition strength was reproducibly correlated with increased -RC_bray_ stability. The majority of associations between network characteristics and stability estimates were confirmed using external sources. Among the 22 associations for the Prev enterotype and 46 for Rum, there were 17 and 21, respectively, that exhibited significant associations with consistent directions in at least one external database, while 0 and 7 had the opposite direction (additionally, 2 associations in the Rum enterotype exhibited conflicting directions within the external databases themselves) (Fig. 4, Supplementary Figure 5). This suggests that the constructed models recapitulate important associations between stability and specific features of the community interaction network.

## 3 Materials and methods

### 3.1 Public data collection and processing

For the purpose of meta-analysis, we collected microbiome datasets that met specific criteria. These criteria included having at least 2 samples per individual with a time interval between sampling ranging from 1 day to 2 months (63 days). We focused on microbiome profiles obtained using 16S rRNA gene sequencing. The datasets we collected consisted of longitudinal studies conducted without external interventions, as well as dietary interventional studies.

In total, we obtained data from 9 studies. Relevant information about each study is presented in Table 1. We only included microbiome samples collected within the first 2 months for studies lasting longer than 2 months (PROTEIN, FMTD). For FMTD study, we chose a two-month interval with the highest sample density for each subject.

**Table 1:** Studies included in meta-analysis.

Abbreviation	Study design	Number of subjects	Time points per individual	Data availability
		(initial / taken in meta-analysis)		
TEMP[56]	Time series without interventions	20 / 20	∼30 / ∼30	Online supplementary table - genus-level relative abundance
RS2[57]	Consumption of resistant starch (RS) from potato for 1 week	20 / 20	∼8 / ∼8	PRJNA306884
PROTEIN[58]	Red meat, white meat and non-meat diets, each 4 weeks with 2 weeks washout periods between them	109 / 96	4 / 2	PRJNA498128
MEAT[59]	Case-control study, test group replace meat with plant-based alternatives for 4 weeks	39 / 38	2 / 2	PRJNA738373
HFD[41]	2 weeks of personalised recommendations towards enhancing fibre intake	215 / 201	2 / 2	PRJEB16344
FMTD[60]	Time series without interventions	90 / 43	2–192 / 2–56	PRJNA544527
FDP[61]	Intake of a product fortified with probiotic for 2 months	150 / 129	2 / 2	PRJEB26974
F3[62]	Case-control study with three test groups and 1 placebo group. Test groups: RS from potato, maize and chicory root for 2 weeks	174 / 174	∼8 / ∼8	PRJNA428736
FIBER[63]	Crossover placebo-controlled intake of inulin-type fructan prebiotic for 3 weeks	34 / 34	4 / 4	PRJNA414683

For all studies but the TEMP [56], we conducted data processing starting from raw sequences. The sequences were downloaded from the NCBI Sequence Read Archive (Table 1). The FIBER study was the only one focused on the V3-V4 regions of 16S rRNA, while all the others had V4 region sequenced. Therefore, for the FIBER dataset, the V4 region sequences were extracted with cutadapt software using 515F/806R [64] primer sequences [65]. Abundance tables were obtained in two steps: denoising with DADA2 [66] implemented in QIIME2 [67] pipeline to obtain a table of amplicon sequence variants (ASV) abundances and their taxonomic assignment with the RDP classifier trained on rdp_train_set_16.fa [68]. Only the ASVs resolved by the classifier at the level of the genus were used for subsequent analysis. For the TEMP dataset, we used publicly available abundance tables at the genus level. The algorithm for abundance table generation in the original TEMP article was comparable to ours and also included denoising with DADA2 along with the taxonomic assignment via RDP [56].

The samples with less than 3000 reads were filtered out from the obtained abundance tables. If there was only one sample per subject left, it was also discarded. Additionally, for the HFD and FDP studies, we excluded the samples with abnormal composition, such as those dominated by *Enterobacteriaceae* members. The final list of samples included in the meta-analysis is provided in Supplementary Table 1. We also conducted taxa filtration, retaining only genera observed in ≥ 3 studies and with an abundance > 1% in at least 2 consecutive time points of at least 100 subjects. The final abundance table included 3512 samples and 41 genera.

The filtered abundance tables were subject to enterotyping using the Dirichlet multinomial mixture models (R package DirichletMultinomial) [69], [70] yielding 4 clusters. The clusters were named according to the criteria listed in the paper by Valles-Colomer et al. [45]. For each subject, all of his/her samples were assigned the most prevalent enterotype for that subject. The abundance tables obtained for each enterotype were filtered again to discard rare taxa. Firstly, the taxa were sorted by the number of subjects with a taxon abundance of > 1% in at least 2 consecutive time points. We selected the number of top taxa from the sorted list equal to the square root of the number of samples in the enterotype, which is the recommended criteria of taxa/sample relationship for fitting models of community dynamics [25]. The taxa detected in < 25 subjects in the sorted list according to the initial selection were also discarded.

The alpha diversity was calculated at the genus level after rarefication to the minimum number of reads per sample using Shannon metric.

### 3.2 Gut microbiome dynamics inference

To describe the dynamics of the gut microbiome, we chose the compositional Lotka-Volterra (cLV) model [15]. Unlike the classic generalised Lotka-Volterra (gLV) model, cLV works with relative abundances, making it more suitable for compositional microbiome data. The parameters characterising microbial community dynamics in the cLV model are the relative interaction matrix *A*, the relative bacterial growth rates vector *g*, and the matrix of external effects' relative influences on bacterial taxa *B*. We trained the aforementioned model using the code from the original cLV article [15] for every enterotype separately (Rum, Prev, Bact1, Bact2), as well as for unstratified data. During the training process, we took into account the time interval between consecutive samples and each of the 10 interventions from our data as independent external effects. Elastic net regression was chosen for regularisation during the model training. To evaluate the accuracy of model prediction, we conducted cross-validation for each of 5 model types. To optimise computation time, we used the following cross-validation scheme:

- First, we divided all subjects from the data in a 25:75 ratio while balancing the interventions between the groups (train_test_split function from scikit-learn package [71], only the interventions with >3 subjects were considered);
- Regularisation parameters training, being the most resource-intensive step, was performed once for each model type using the 75% group;
- Cross-validation was performed in a leave-one-out manner using the 75% group together with all-except-one subjects from the 25% group as the training data.

The quality of the models was assessed using three metrics to compare predicted and true relative abundances: Root Mean Square Error (RMSE), Root Mean Squared Logarithmic Error (RMSLE) and Aitchison distance. The RMSLE and Aitchison distance were calculated after replacing all zeros in relative abundances with a 10^-5^ pseudocount. To evaluate the prediction quality of the trained cLV models, we compared them to static models that assumed no change in the microbiome composition of subjects over time. This comparison was done using the Wilcoxon rank sum test. The final relative interaction matrices and relative growth rate vectors were obtained using full datasets for each of the 5 model types. To estimate the reproducibility of interaction matrices, we trained all of our 5 models on 50% of each dataset 20 times and calculated intra-enterotype Pearson correlations over the obtained series of interaction matrices. Since good prediction quality was only achieved for the Rum and Prev enterotypes, all further analyses based on cLV results were restricted to individuals from these enterotypes.

### 3.3 External databases for relative interactions validation

The relative interaction coefficients between genera from cLV were compared to three external interaction networks. These networks include the NJC19 network, a literature-curated network for the mammalian gut microbiome [50]; the GutCP network, a published network for school-aged children's microbiome obtained using the GutCP algorithm [49]; and a network constructed for enterotype medoids using AGORA genome-scale metabolic models (GSMMs) with the MICOM algorithm [51], [72]. The NJC19 and GutCP networks were obtained directly from the corresponding publications as tables, which included information about microbes, metabolites, and types of metabolic activity (import or export). For the MICOM algorithm, we applied it to the enterotype medoids from our data, utilising the average Western diet (western_diet_gut.qza implemented in the package) as the growth medium. The resulting table "exchange_fluxes" was used for further calculations.

For each of the three tables, we calculated competition and cross-feeding indices, making slight modifications to a previously suggested approach [52]. To calculate the cross-feeding index for each pair of genera, we determined the proportion of metabolites that are simultaneously exported by species from one genus and imported by species from the other genus. To obtain the competition index, we calculated the proportion of metabolites simultaneously imported by species from two genera. We then conducted a comparison of these indices with the relative interaction strength from cLV using linear regression.

### 3.4 Obtaining microbial composition in steady state for each individual

To obtain microbial compositions in steady states, we employed a combination of *in silico* simulations and analytical calculations. First, we generated one community dynamics model per individual by selecting equations related to genera having non-zero baseline relative abundances. Subsequently, we simulated the dynamics of each individual's baseline microbiome profile over a large time interval (e.g., 100,000 days) using the code provided in the original cLV article [15]. During the simulations, we controlled the saturation of beta diversity reduction between profiles with a one-day difference (mean daily Bray-Curtis dissimilarity at the end of the simulation was 1⋅10^-3^ ± 2⋅10^-3^). Next, we obtained analytical solutions for the equilibrium points by solving the cLV equation (described in the appendix of the original cLV article [15]) for each individual's model. We observed that in each case, the simulation results converged to one of the analytical solutions, and we selected this solution as the steady state for the individual (mean Bray-Curtis dissimilarity between simulation results and corresponding analytical solutions was 8⋅10^-5^ ± 4⋅10^-5^). To optimise computational time, we looked for the closest analytical solutions among those with zero abundance of genera, which had an abundance of less than 1⋅10^-5^ at the end of the simulations. The above-described calculations were conducted using Python v. 3.9. Analytical solutions were obtained using scipy.solve function [71].

### 3.5 Stability measures: calculating and deriving cross-correlations

All further calculations were conducted in R version 4.2.2. Using the collected data, trained models and information about steady states, we calculated 4 ecological and 9 observational stability measures. The following ecological estimates were used to calculate stability for each individual:

- Local stability was measured as multiplied by − 1 the maximum real eigenvalues part of the dynamical systems’ Jacobian matrix for each individual’s steady state (-R(λ_max_));
- Distance from the baseline sample to each individual’s steady state was measured using Bray-Curtis dissimilarity and Aitchison distance (StP(1-BC) and StP(-Ait), respectively);
- Robustness was defined as the average number of genera that have to be removed from the community to cause the loss of 50% of initial community richness [11] (Rob0.5). It was estimated via in silico simulations. Firstly, for each baseline sample we excluded from 1 to N/2 random microbes, each number of microbes was excluded 10 times (N - total number of microbes). For all obtained microbiome profiles from this procedure, we estimated a composition in a steady state as described in the previous section. The mean number of microbes with zero abundance in the steady state was calculated across 10 iterations for each number of initially removed genera. Boundary estimation for zero abundance was 1⋅10^-5^. The obtained experimental dependence was then approximated by a polynomial function to determine the number of initially removed genera causing secondary extinction of N/2 microbes.

Ecological modelling stability measures were obtained only for individuals from Rum and Prev enterotypes (N = 413).

The observational stability estimates included measurements and predictions of inverse microbiome change. For measurements we used 1st and 2nd time points for each individual and the following metrics:

- Inverse Bray-Curtis dissimilarity (1-BC);
- Inverse Weighted Unifrac distance [53] (1-WUni);
- Pearson's correlation coefficient (Cor);
- Inverse Euclidean distance after log transformation (1-Euc(log));
- Inverse Aitchison distance [54] (-Ait);
- Inverse Jaccard index (1-Jaccard);
- Inverse Raup–Crick metric based on Bray–Curtis dissimilarity using all samples from the same enterotype as a context [55] (-RC_bray_).

The inverse values were calculated as a subtraction of beta diversity from 1 for measures ranging from 0 to 1 (Bray-Curtis, Weighted Unifrac, Euclidean, Jaccard) and beta diversity multiplied by − 1 for other measures (Aitchison, Raup–Crick).

For predictions we used baseline samples. For each sample, we calculated inverse response potential [38] using the baseline samples from all other individuals from the same enterotype as a context. Inverse response potential was calculated using the following metrics:

- Inverse Bray-Curtis dissimilarity (P(1-BC));
- Inverse Aitchison distance [54] (P(-Ait)).

All observational stability measures except for the inverse Weighted Unifrac distance were derived for all individuals (N = 755), while the latter was derived with the exclusion of the TEMP cohort (yielding N = 735).

All estimated stability measures were adjusted for time interval and intervention type between 1st and 2nd time points, as well as for the baseline Shannon alpha diversity using linear regression. Adjustment for alpha diversity has been included to account for the computational dependency between alpha and beta diversity [38]. The model residuals were then compared with each other using Pearson correlation (R function cor.test [73]). We compared measures for Rum and Prev enterotypes separately. Correction for multiple comparisons was performed using the Benjamini–Hochberg method.

We carried out hierarchical clustering (using complete linkage) based on the obtained correlations and analysed the dependence of stability measures in each of the obtained clusters with the baseline taxa abundances separately for each enterotype. The taxa abundances after the centred log ratio (clr) transformation were used. The analysis was conducted using a linear model with the adjustment for time interval and intervention type between 1st and 2nd time points, as well as for the baseline Shannon alpha diversity. Correction for multiple comparisons was performed using the Benjamini–Hochberg method.

### 3.6 Associations between stability and relative interaction matrix characteristics

All the aforementioned stability metrics were compared to the characteristics of relative interaction matrices obtained using cLV for the Rum and Prev enterotypes. For each individual, only the interactions between the taxa with non-zero baseline abundance were considered. The following parameters were calculated:

- Connectance (*C*) - the average number of edges per node (*A*_*ij*_ and *A*_*ji*_ were considered as separate edges);
- Heterogeneity of node degrees (*H*) - the variance in the number of edges per node;
- Interaction strength (*S*) - the mean absolute value of non-zero interactions;
- Average competition strength (*S*_*c*_) - the average absolute value of mutually negative relative interactions (considering all interactions that were not mutually negative as having a zero value);
- Average mutualism strength (*S*_*m*_) - the average value of mutually positive relative interactions (considering all interactions that were not mutually positive as having a zero value);
- Coefficient characterising the network decomposition (*b*) - the value calculated using the method proposed by Carpentier et al. [11] using the information about the number of edges and number of nodes.

Associations between these characteristics and stability measures were calculated similarly to the cross-correlation between stability estimates described above.

We conducted further validation of this analysis by using external databases that have been previously described: GutCP [49], MICOM [51] and NJC19 [50]. To refine our analysis, we excluded metabolites that appeared in > 70% of genera pairs (specifically, "Acetate" in NJC19 and "Isovalerate" in GutCP) and recalculated the cross-feeding and competition indices. After filtering out taxa with zero baseline abundances, we constructed interaction networks for each subject using the resulting indices. To increase the number of intersections between the microbiome profiles and interaction networks, we applied filtration using the abundance table that was previously prepared for the "unstratified" analysis option (containing 41 genera). An interaction between two genera was considered to exist if at least one of the cross-feeding or competition indices was non-zero. The calculations for *C*, *H*, and *b* values were performed following the same methodology as described above for the relative interaction network. *S*_*m*_ and *S*_*c*_ were estimated as average cross-feeding and competition indices, respectively. To calculate *S*, we first obtained the mean value between the cross-feeding and competition indices for each edge and then calculated the average of the non-zero values.

## 4 Discussion

The importance of integrating theoretical ecology and microbiome data analysis fields has been emphasised in several review papers [74], [75], [76]. These areas of knowledge have historically developed independently, and only a few works have successfully integrated them, leading to a significant deepening of our understanding of microbial ecology [7], [13], [14], [23], [76]. Our work is one of the first to integrate these two approaches in the context of human gut microbiome stability through a meta-analysis.

One of the major challenges in applying microbial ecology approaches to the analysis of gut microbiome data is the limited availability of time series with the number of samples sufficient to accurately infer the dynamics of a large number of taxa. To address this issue, we made several assumptions. Firstly, we assumed that microbial interactions, growth rates, and responses to external stimuli are universal across hosts. Although this assumption allowed us to significantly expand the training sample size, it oversimplifies the real situation, as the internal factors of the host organism, lifestyle and nutrition patterns can introduce significant variation in the parameters. Another source of variability could be due to considerable gene content variability across the taxa represented by the same 16S rRNA read sequence. However, previous observations suggest the universality of at least some of the systems' parameters between hosts. In particular, the dissimilarity-overlap analysis [28] demonstrated that as the overlap between communities of different individuals increases, the dissimilarity between communities decreases. This implies the presence of universal dynamics, although it is not a sufficient condition for it [26], [28]. To at least partly take into account the inter-subject variability, we stratified the data into enterotypes - thus assuming universality only among the subjects with similar microbiomes. Although enterotypes do not serve as a crucial foundation for all our work, we have used them to divide feature spaces into more manageable units that can be more accurately described by mathematical models. Our study revealed that stratification into enterotypes improved the prediction quality when compared to the static model, but only for 2 out of 4 enterotypes - Rum and Prev. This partially supports the assumption of microbiome dynamics' universality for these two enterotypes. However, other reasons are not excluded, including the uneven distribution of time intervals and interventions between enterotypes in our data.

The next important generalisation is the use of genus-level taxon abundances during modelling. While it would be preferable to perform modelling at a more detailed species level, the limitation of 16S rRNA gene sequencing is the inability to recognize many microbes at the level of species. Particularly, it has been demonstrated that for 16S data, modelling via GSMMs showed similar performance quality for species and genus levels [51]. Currently, more and more studies are using whole genome sequencing (WGS) for taxonomic profiling, which allows for obtaining more detailed microbiome compositions. Therefore, it is likely that a sufficient volume of publicly available WGS time-series data will soon be accumulated to conduct an analysis similar to that described above using microbiome compositions at the level of species.

Another thing of note was that we carried out a rather rigorous filtration of rare taxa, resulting in 20–41 genera =per enterotype. Although rare taxa can have a significant impact on the community ecology, our study was limited by the quantity of data collected and the challenges with handling rare taxa in microbiome data. Given the compositional nature of microbiome data, we lack information on absolute abundances and face difficulties in distinguishing between biological and technical zeros [77]. In the context of compositional data analysis, zeros are typically substituted with pseudo-counts. While this procedure does not adversely affect data with moderate sparsity, it can considerably amplify the noise level in datasets containing numerous rare taxa. However, the accumulation of a larger number of microbiome time series and increase of sequencing coverage will allow more detailed data stratification and account for rarer taxa without increasing the data sparsity.

Lastly, we would like to mention two assumptions related to uncontrolled external factors and the maintenance of ecosystem parameters over a 2-month period. These assumptions arise from the analysis of real stool samples obtained from human subjects. Unlike in vitro and model animal studies, human subjects are consistently exposed to a wide range of external influences, making it challenging to conduct large-scale experiments under strictly controlled conditions. Additionally, unlike in vitro experiments, the frequency of stool sample collection is limited, necessitating extended time intervals to gather comprehensive time-series data.

Although the assumptions described above simplify the dynamics of the microbial community, we consider the results to be a significant advancement in integrating theoretical ecology and microbiome data analysis. Our study has shown a strong agreement between stability measured through observational approaches, employing metrics with log transformation, and ecology-guided local stability. Log transformation not only balances the contributions of high and low-abundant taxa but also allows us to measure microbiome change in a compositional manner. As postulated in compositional analysis, the calculation of distances should take into account the ratio, rather than the difference between taxa abundances [54], [78]. We also revealed intriguing positive correlations between response potential and distances from steady states. Both approaches involve assessing the sample's position on the stability landscape. However, one method implies calculating the distance to the mathematically determined equilibrium point, while the other method considers the distances to multiple samples from different individuals [38].

Our findings provide further confirmation of the established trade-off between robustness and local stability, mediated by opposite dependencies of these metrics on the coefficient characterising network decomposition [11]. Through the analysis of the relative interaction network characteristics, we discovered that higher connectance and heterogeneity in the collected microbiome samples are associated with lower average interaction strength. This observation aligns well with the complexity-stability trade-off paradigm [5]. Several intriguing dependencies were revealed for the -RC_bray_ stability metric. The RC_bray_ dissimilarity compares the differences between the analysed communities with those between randomly generated ones. Therefore, this metric allows us to suggest what type of processes shape and maintain the communities. When the metric is close to 0, it indicates that stochastic processes play a significant role, while values near 1 or − 1 indicate deterministic processes that give an advantage to different species in the case of 1 and to similar ones in the case of − 1 [79]. In our data, the -RC_bray_ stability values ranged from − 1–0 for most of the samples (97%). This implies that communities with lower -RC_bray_ stability values underwent changes influenced by deterministic processes, providing advantages to different species at different time points. Our meta-analysis showed that such communities were less robust (less resistant to species loss) and also had higher connectance, heterogeneity and lower competition strength. Correlations observed between stability metrics and network characteristics were validated by examining networks obtained from external sources, reinforcing the robustness of the obtained results. This indicates that the parameters derived from the relative interaction network are likely indicative of the actual interactions within the microbial community.

As for the roles of individual microbes in the community stability, we found that the communities with higher abundances of enterotype driver taxa (*Prevotella*, *Bacteroides*, *Faecalibacterium*, *Roseburia* and *Blautia*) are more resistant to species loss (the listed taxa are positively associated with a cluster of stability metrics that includes robustness). At the same time, such communities diverge faster from the steady state under small disturbances (the same taxa are negatively associated with a cluster of stability metrics that includes local stability). Depletion of some of these taxa (*Faecalibacterium*, *Prevotella* and *Roseburia*) was reported for various diseases [80], [81], [82], [83], [84], [85], [86]. In the cases of *Faecalibacterium* and *Roseburia*, this has primarily been attributed to their involvement in the production of butyrate, which has multiple positive effects on intestinal health [87]. Contrarily, among the microbes elevated in communities less resistant to species loss and more locally stable, there were a number of opportunistic bacteria linked to disorders (*Collinsella*, *Oscillibacter*). *Collinsella* was previously associated with host diet [88], [89], [90], multiple diseases and unfavourable metabolic markers [88], [90], [91], [92], [93], [94]. One of the possible mechanisms is that the members of this genus promote gut permeability and production of pro-inflammatory cytokines [86]. Interestingly, *Oscillibacter* has also been associated with pro-inflammatory cytokines and intestinal permeability [95], as well as several disease states [81], [96], [97]. These findings highlight the importance of considering the specific properties of the selected stability metrics when studying factors that contribute to the stability of the microbiome in health and disease.

In our study, we have focused on healthy individuals, but an important future direction is to extend the proposed methodology to diseased individuals. Currently, most studies to investigate the relationship between the microbiome and specific diseases are cross-sectional and provide information about the microbial taxa that are over- or underrepresented at a single time point compared to the healthy controls. However, in some cases, differences from healthy individuals are rather manifested in the dynamics of the microbiome [14], [36], [98]. Shifting from cross-sectional studies to time series will allow us to expand our understanding of the microbiome’s role in various disorders. One of the crucial steps to achieve this goal is developing adequate methods for microbiome time series data processing. In this study, we compared approaches from different fields to inspecting one of the most important dynamic characteristics of the microbiome - its stability. Several studies have shown that the stability of the gut microbiome is decreased in subjects with particular diseases compared to healthy ones [14], [36], [98], [99]. At the same time, there can exist chronic dysbiotic changes of the microbiome characterised by high stability, which are extremely difficult to correct [1], [44]. The ecosystem parameters contributing to stability shifts in such cases are still to be identified. Our study, which showed consistency between stability measured through observational and through ecological modelling approaches, indicates that modelling can be one of the promising ways to investigate this question with the accumulation of more data. It can help to identify specific taxa or interaction types in the community that affect stability in diseases and also allow for the preliminary evaluation of the effectiveness of certain types of preventive and therapeutic interventions, such as probiotics or prebiotics intake.

## 5 Conclusions

We successfully integrated observational and ecological modelling approaches to provide a comprehensive understanding of human gut microbiome stability. By employing compositionality-aware methods, we revealed that the stability of the microbial community is influenced by the characteristics of the network of relative interactions between microbes. Additionally, we validated our findings by comparing them with networks obtained from external sources. The combination of theoretical ecology, statistical analysis, and observational data allowed us to effectively model microbiome dynamics, offering insights into the relationship between the human microbiome, health, and disease, as well as its connection to nutrition and population-wide surveys. This approach holds promise for further investigations into the complex dynamics of the human gut microbiome and its implications for various aspects of human health.

## Funding

This work was supported by the 10.13039/501100006769Russian Science Foundation (project #22–24–00683).

## CRediT authorship contribution statement

Anastasia Revel-Muroz: Methodology, Investigation, Formal analysis, Data Curation, Visualization, Writing – original draft. Mikhail Akulinin: Investigation, Formal analysis, Data Curation. Polina Shilova: Investigation, Formal analysis, Data Curation. Alexander Tyakht: Conceptualization, Methodology, Supervision, Writing – original draft, Writing - review & editing. Natalia Klimenko: Conceptualization, Methodology, Supervision, Investigation, Formal analysis, Data Curation, Visualization, Writing – original draft, Writing - review & editing.

## Declaration of Generative AI and AI-assisted technologies in the writing process

During the preparation of this work the authors used ChatGPT in order to improve readability in English and correct grammatical errors. After using the service, the authors reviewed and edited the content as needed and take full responsibility for the content of the publication.

## Declaration of Competing Interest

The authors declare no competing interests.

## Data Availability

In this study, we re-analysed raw data from the previously published datasets (see Table 1).

## References

[1] Fassarella M., Blaak E.E., Penders J., Nauta A., Smidt H., Zoetendal E.G. (2021). Gut microbiome stability and resilience: elucidating the response to perturbations in order to modulate gut health. Gut.

[2] Allesina S., Tang S. (2012). Stability criteria for complex ecosystems. Nature.

[3] Butler S., O’Dwyer J.P. (2018). Stability criteria for complex microbial communities. Nat Commun.

[4] Stone L. (2020). The stability of mutualism. Nat Commun.

[5] May R.M. (1972). Will a large complex system be stable?. Nature.

[6] Rohr R.P., Saavedra S., Bascompte J. (2014). Ecological networks. On the structural stability of mutualistic systems. Science.

[7] Coyte K.Z., Schluter J., Foster K.R. (2015). The ecology of the microbiome: networks, competition, and stability. Science.

[8] Qian J.J., Akçay E. (2020). The balance of interaction types determines the assembly and stability of ecological communities. Nat Ecol Evol.

[9] Dubinkina V., Fridman Y., Pandey P.P., Maslov S. (2019). Multistability and regime shifts in microbial communities explained by competition for essential nutrients. Elife.

[10] Dunne J.A., Williams R.J., Martinez N.D. (2002). Network structure and biodiversity loss in food webs: robustness increases with connectance. Ecol Lett.

[11] Carpentier C., Barabás G., Spaak J.W., De, Laender F. (2021). Reinterpreting the relationship between number of species and number of links connects community structure and stability. Nat Ecol Evol.

[12] Grilli J., Adorisio M., Suweis S., Barabás G., Banavar J.R., Allesina S. (2017). Feasibility and coexistence of large ecological communities. Nat Commun.

[13] Stein R.R., Bucci V., Toussaint N.C., Buffie C.G., Rätsch G., Pamer E.G. (2013). Ecological modeling from time-series inference: insight into dynamics and stability of intestinal microbiota. PLoS Comput Biol.

[14] Gibson T.E., Kim Y., Acharya S., Kaplan D.E., DiBenedetto N., Lavin R. (2021). Intrinsic instability of the dysbiotic microbiome revealed through dynamical systems inference at scale. bioRxiv.

[15] Joseph T.A., Shenhav L., Xavier J.B., Halperin E., Pe’er I. (2020). Compositional Lotka-Volterra describes microbial dynamics in the simplex. PLoS Comput Biol.

[16] Liu H., Liao C., Wu L., Tang J., Chen J., Lei C. (2022). Ecological dynamics of the gut microbiome in response to dietary fiber. ISME J.

[17] Gao X., Huynh B.-T., Guillemot D., Glaser P., Opatowski L. (2018). Inference of Significant Microbial Interactions From Longitudinal Metagenomics Data. Front Microbiol.

[18] Li J., Shen X., Li Y. (2021). Modeling the temporal dynamics of gut microbiota from a local community perspective. Ecol Model.

[19] Rao C., Coyte K.Z., Bainter W., Geha R.S., Martin C.R., Rakoff-Nahoum S. (2021). Multi-kingdom ecological drivers of microbiota assembly in preterm infants. Nature.

[20] Fisher C.K., Mehta P. (2014). Identifying keystone species in the human gut microbiome from metagenomic timeseries using sparse linear regression. PLoS One.

[21] Ho P.-Y., Good B.H., Huang K.C. (2022). Competition for fluctuating resources reproduces statistics of species abundance over time across wide-ranging microbiotas. Elife.

[22] Gao F., Guo R., Ma Q., Li Y., Wang W., Fan Y. (2022). Stressful events induce long-term gut microbiota dysbiosis and associated post-traumatic stress symptoms in healthcare workers fighting against COVID-19. J Affect Disord.

[23] Bucci V., Tzen B., Li N., Simmons M., Tanoue T., Bogart E. (2016). MDSINE: microbial dynamical systems inference engine for microbiome time-series analyses. Genome Biol.

[24] Shaw L.P., Bassam H., Barnes C.P., Walker A.S., Klein N., Balloux F. (2019). Modelling microbiome recovery after antibiotics using a stability landscape framework. ISME J.

[25] Gonze D., Coyte K.Z., Lahti L., Faust K. (2018). Microbial communities as dynamical systems. Curr Opin Microbiol.

[26] Bashan A., Gibson T.E., Friedman J., Carey V.J., Weiss S.T., Hohmann E.L. (2016). Universality of human microbial dynamics. Nature.

[27] Cao H.-T., Gibson T.E., Bashan A., Liu Y.-Y. (2017). Inferring human microbial dynamics from temporal metagenomics data: pitfalls and lessons. Bioessays.

[28] Yonatan Y., Amit G., Friedman J., Bashan A. (2022). Complexity-stability trade-off in empirical microbial ecosystems. Nat Ecol Evol.

[29] Gibson T.E., Carey V., Bashan A., Hohmann E.L., Weiss S.T., Liu Y.-Y. (2017). On the stability landscape of the human gut microbiome: implications for microbiome-based therapies. bioRxiv.

[30] Gibson T.E., Bashan A., Cao H.-T., Weiss S.T., Liu Y.-Y. (2016). On the Origins and Control of Community Types in the Human Microbiome. PLoS Comput Biol.

[31] Mehta R.S., Abu-Ali G.S., Drew D.A., Lloyd-Price J., Subramanian A., Lochhead P. (2018). Stability of the human faecal microbiome in a cohort of adult men. Nat Microbiol.

[32] Byrd A.L., Liu M., Fujimura K.E., Lyalina S., Nagarkar D.R., Charbit B. (2021). Gut microbiome stability and dynamics in healthy donors and patients with non-gastrointestinal cancers. J Exp Med.

[33] Faith J.J., Guruge J.L., Charbonneau M., Subramanian S., Seedorf H., Goodman A.L. (2013). The long-term stability of the human gut microbiota. Science.

[34] Flores G.E., Caporaso J.G., Henley J.B., Rideout J.R., Domogala D., Chase J. (2014). Temporal variability is a personalized feature of the human microbiome. Genome Biol.

[35] Johnson A.J., Vangay P., Al-Ghalith G.A., Hillmann B.M., Ward T.L., Shields-Cutler R.R. (2019). Daily sampling reveals personalized diet-microbiome associations in humans. Cell Host Microbe.

[36] Frost F., Kacprowski T., Rühlemann M., Pietzner M., Bang C., Franke A. (2021). Long-term instability of the intestinal microbiome is associated with metabolic liver disease, low microbiota diversity, diabetes mellitus and impaired exocrine pancreatic function. Gut.

[37] Shade A., Caporaso J.G., Handelsman J., Knight R., Fierer N. (2013). A meta-analysis of changes in bacterial and archaeal communities with time. ISME J.

[38] Klimenko N.S., Odintsova V.E., Revel-Muroz A., Tyakht A.V. (2022). The hallmarks of dietary intervention-resilient gut microbiome. NPJ Biofilms Micro.

[39] David L.A., Materna A.C., Friedman J., Campos-Baptista M.I., Blackburn M.C., Perrotta A. (2014). Host lifestyle affects human microbiota on daily timescales. Genome Biol.

[40] Dethlefsen L., Relman D.A. (2011). Incomplete recovery and individualized responses of the human distal gut microbiota to repeated antibiotic perturbation. Proc Natl Acad Sci USA.

[41] Klimenko N., Tyakht A., Popenko A., Vasiliev A., Altukhov I., Ischenko D. (2018). Microbiome responses to an uncontrolled short-term diet intervention in the frame of the citizen science project. Nutrients.

[42] Liu Z., de Vries B., Gerritsen J., Smidt H., Zoetendal E.G. (2020). Microbiome-based stratification to guide dietary interventions to improve human health. Nutr Res.

[43] Lozupone C.A., Stombaugh J.I., Gordon J.I., Jansson J.K., Knight R. (2012). Diversity, stability and resilience of the human gut microbiota. Nature.

[44] Sommer F., Anderson J.M., Bharti R., Raes J., Rosenstiel P. (2017). The resilience of the intestinal microbiota influences health and disease. Nat Rev Microbiol.

[45] Valles-Colomer M., Falony G., Darzi Y., Tigchelaar E.F., Wang J., Tito R.Y. (2019). The neuroactive potential of the human gut microbiota in quality of life and depression. Nat Microbiol.

[46] Vandeputte D., Kathagen G., D’hoe K., Vieira-Silva S., Valles-Colomer M., Sabino J. (2017). Quantitative microbiome profiling links gut community variation to microbial load. Nature.

[47] Vieira-Silva S., Falony G., Belda E., Nielsen T., Aron-Wisnewsky J., Chakaroun R. (2020). Statin therapy is associated with lower prevalence of gut microbiota dysbiosis. Nature.

[48] McInnes L., Healy J., Melville J. UMAP: Uniform Manifold Approximation and Projection for Dimension Reduction. arXiv [statML] 2018.

[49] Goyal A., Wang T., Dubinkina V., Maslov S. (2021). Ecology-guided prediction of cross-feeding interactions in the human gut microbiome. Nat Commun.

[50] Lim R., Cabatbat J.J.T., Martin T.L.P., Kim H., Kim S., Sung J. (2020). Large-scale metabolic interaction network of the mouse and human gut microbiota. Sci Data.

[51] Diener C., Gibbons S.M., Resendis-Antonio O. (2020). MICOM: metagenome-scale modeling to infer metabolic interactions in the gut microbiota. mSystems.

[52] Levy R., Borenstein E. (2013). Metabolic modeling of species interaction in the human microbiome elucidates community-level assembly rules. Proc Natl Acad Sci USA.

[53] Lozupone C., Knight R. (2005). UniFrac: a new phylogenetic method for comparing microbial communities. Appl Environ Microbiol.

[54] Aitchison J. (1982). The Statistical Analysis of Compositional Data. J R Stat Soc Ser B Stat Method.

[55] Stegen J.C., Lin X., Fredrickson J.K., Chen X., Kennedy D.W., Murray C.J. (2013). Quantifying community assembly processes and identifying features that impose them. ISME J.

[56] Vandeputte D., De Commer L., Tito R.Y., Kathagen G., Sabino J., Vermeire S. (2021). Temporal variability in quantitative human gut microbiome profiles and implications for clinical research. Nat Commun.

[57] Venkataraman A., Sieber J.R., Schmidt A.W., Waldron C., Theis K.R., Schmidt T.M. (2016). Variable responses of human microbiomes to dietary supplementation with resistant starch. Microbiome.

[58] Lang J.M., Pan C., Cantor R.M., Tang W.H.W., Garcia-Garcia J.C., Kurtz I. (2018). Impact of individual traits, saturated fat, and protein source on the gut microbiome. MBio.

[59] Toribio-Mateas M.A., Bester A., Klimenko N. (2021). Impact of plant-based meat alternatives on the gut microbiota of consumers: a real-world study. Foods.

[60] Poyet M., Groussin M., Gibbons S.M., Avila-Pacheco J., Jiang X., Kearney S.M. (2019). A library of human gut bacterial isolates paired with longitudinal multiomics data enables mechanistic microbiome research. Nat Med.

[61] Volokh O., Klimenko N., Berezhnaya Y., Tyakht A., Nesterova P., Popenko A. (2019). Human gut microbiome response induced by fermented dairy product intake in healthy volunteers. Nutrients.

[62] Baxter N.T., Schmidt A.W., Venkataraman A., Kim K.S., Waldron C., Schmidt T.M. (2019). Dynamics of human gut microbiota and short-chain fatty acids in response to dietary interventions with three fermentable fibers. MBio.

[63] Healey G., Murphy R., Butts C., Brough L., Whelan K., Coad J. (2018). Habitual dietary fibre intake influences gut microbiota response to an inulin-type fructan prebiotic: a randomised, double-blind, placebo-controlled, cross-over, human intervention study. Br J Nutr.

[64] Hugerth L.W., Wefer H.A., Lundin S., Jakobsson H.E., Lindberg M., Rodin S. (2014). DegePrime, a program for degenerate primer design for broad-taxonomic-range PCR in microbial ecology studies. Appl Environ Microbiol.

[65] Martin M. (2011). Cutadapt removes adapter sequences from high-throughput sequencing reads. EMBnet J.

[66] Callahan B.J., McMurdie P.J., Rosen M.J., Han A.W., Johnson A.J.A., Holmes S.P. (2016). DADA2: high-resolution sample inference from Illumina amplicon data. Nat Methods.

[67] Bolyen E., Rideout J.R., Dillon M.R., Bokulich N.A., Abnet C.C., Al-Ghalith G.A. (2019). Reproducible, interactive, scalable and extensible microbiome data science using QIIME 2. Nat Biotechnol.

[68] Wang Q., Garrity G.M., Tiedje J.M., Cole J.R. (2007). Naive Bayesian classifier for rapid assignment of rRNA sequences into the new bacterial taxonomy. Appl Environ Microbiol.

[69] Holmes I., Harris K., Quince C. (2012). Dirichlet multinomial mixtures: generative models for microbial metagenomics. PLoS One.

[70] Morgan M. DirichletMultinomial for Clustering and Classification of Microbiome Data n.d. https://bioconductor.statistik.tu-dortmund.de/packages/3.10/bioc/vignettes/DirichletMultinomial/inst/doc/DirichletMultinomial.pdf (Accessed 6 May 2023).

[71] Pedregosa F., Varoquaux G., Gramfort A., Michel V., Thirion B., Grisel O. (2011). Scikit-learn: Machine Learning. Python J Mach Learn Res.

[72] Magnúsdóttir S., Heinken A., Kutt L., Ravcheev D.A., Bauer E., Noronha A. (2017). Generation of genome-scale metabolic reconstructions for 773 members of the human gut microbiota. Nat Biotechnol.

[bib73] 73Foundation for Statistical Computing RR. R: a language and environment for statistical computing. RA Lang Environ Stat Comput n.d.

[74] Tipton L., Darcy J.L., Hynson N.A. (2019). A developing symbiosis: enabling cross-talk between ecologists and microbiome scientists. Front Microbiol.

[75] Stenuit B., Agathos S.N. (2015). Deciphering microbial community robustness through synthetic ecology and molecular systems synecology. Curr Opin Biotechnol.

[76] McNally L., Brown S.P. (2016). Microbiome: ecology of stable gut communities. Nat Microbiol.

[77] Gloor G.B., Macklaim J.M., Pawlowsky-Glahn V., Egozcue J.J. (2017). Microbiome datasets are compositional: and this is not optional. Front Microbiol.

[78] Odintsova V.E., Klimenko N.S., Tyakht A.V. (2022). Approximation of a microbiome composition shift by a change in a single balance between two groups of taxa. mSystems.

[79] Chase J.M., Kraft N.J.B., Smith K.G., Vellend M., Inouye B.D. (2011). Using null models to disentangle variation in community dissimilarity from variation in α-diversity. Ecosphere.

[80] Nie K., Ma K., Luo W., Shen Z., Yang Z., Xiao M. (2021). Roseburia intestinalis: a beneficial gut organism from the discoveries in genus and species. Front Cell Infect Microbiol.

[81] Gacesa R., Kurilshikov A., Vich Vila A., Sinha T., Klaassen M.A.Y., Bolte L.A. (2022). Environmental factors shaping the gut microbiome in a Dutch population. Nature.

[82] Machiels K., Joossens M., Sabino J., De Preter V., Arijs I., Eeckhaut V. (2014). A decrease of the butyrate-producing species Roseburia hominis and Faecalibacterium prausnitzii defines dysbiosis in patients with ulcerative colitis. Gut.

[83] Jiang S., Xie S., Lv D., Wang P., He H., Zhang T. (2017). Alteration of the gut microbiota in Chinese population with chronic kidney disease. Sci Rep.

[84] Jiang S., Xie S., Lv D., Zhang Y., Deng J., Zeng L. (2016). A reduction in the butyrate producing species Roseburia spp. and Faecalibacterium prausnitzii is associated with chronic kidney disease progression. Antonie Van Leeuwenhoek.

[85] Abbas-Egbariya H., Haberman Y., Braun T., Hadar R., Denson L., Gal-Mor O. (2022). Meta-analysis defines predominant shared microbial responses in various diseases and a specific inflammatory bowel disease signal. Genome Biol.

[86] Chen J., Wright K., Davis J.M., Jeraldo P., Marietta E.V., Murray J. (2016). An expansion of rare lineage intestinal microbes characterizes rheumatoid arthritis. Genome Med.

[87] Hamer H.M., Jonkers D., Venema K., Vanhoutvin S., Troost F.J., Brummer R.-J. (2008). Review article: the role of butyrate on colonic function. Aliment Pharm Ther.

[88] Frost F., Storck L.J., Kacprowski T., Gärtner S., Rühlemann M., Bang C. (2019). A structured weight loss program increases gut microbiota phylogenetic diversity and reduces levels of Collinsella in obese type 2 diabetics: a pilot study. PLoS One.

[89] Gomez-Arango L.F., Barrett H.L., Wilkinson S.A., Callaway L.K., McIntyre H.D., Morrison M. (2018). Low dietary fiber intake increases Collinsella abundance in the gut microbiota of overweight and obese pregnant women. Gut Microbes.

[90] Candela M., Biagi E., Soverini M., Consolandi C., Quercia S., Severgnini M. (2016). Modulation of gut microbiota dysbioses in type 2 diabetic patients by macrobiotic Ma-Pi 2 diet. Br J Nutr.

[91] Ruiz-Limón P., Mena-Vázquez N., Moreno-Indias I., Manrique-Arija S., Lisbona-Montañez J.M., Cano-García L. (2022). Collinsella is associated with cumulative inflammatory burden in an established rheumatoid arthritis cohort. Biomed Pharm.

[92] Karlsson F.H., Fåk F., Nookaew I., Tremaroli V., Fagerberg B., Petranovic D. (2012). Symptomatic atherosclerosis is associated with an altered gut metagenome. Nat Commun.

[93] Astbury S., Atallah E., Vijay A., Aithal G.P., Grove J.I., Valdes A.M. (2020). Lower gut microbiome diversity and higher abundance of proinflammatory genus Collinsella are associated with biopsy-proven nonalcoholic steatohepatitis. Gut Microbes.

[94] Lambeth S.M., Carson T., Lowe J., Ramaraj T., Leff J.W., Luo L. (2015). Composition, diversity and abundance of gut microbiome in prediabetes and type 2 diabetes. J Diabetes Obes.

[95] Lam Y.Y., Ha C.W.Y., Campbell C.R., Mitchell A.J., Dinudom A., Oscarsson J. (2012). Increased gut permeability and microbiota change associate with mesenteric fat inflammation and metabolic dysfunction in diet-induced obese mice. PLoS One.

[96] Kim J.E., Kim H.-E., Park J.I., Cho H., Kwak M.-J., Kim B.-Y. (2020). The association between gut microbiota and uremia of chronic kidney disease. Microorganisms.

[97] Liu Y., Chen L., Liu L., Zhao S.-S., You J.-Q., Zhao X.-J. (2022). Interplay between dietary intake, gut microbiota, and metabolic profile in obese adolescents: Sex-dependent differential patterns. Clin Nutr.

[98] Dinleyici E.C., Martínez-Martínez D., Kara A., Karbuz A., Dalgic N., Metin O. (2018). Time series analysis of the microbiota of children suffering from acute infectious diarrhea and their recovery after treatment. Front Microbiol.

[99] Halfvarson J., Brislawn C.J., Lamendella R., Vázquez-Baeza Y., Walters W.A., Bramer L.M. (2017). Dynamics of the human gut microbiome in inflammatory bowel disease. Nat Microbiol.

