# Supplementary material for: Stability of human gut microbiome: Comparison of ecological modelling and observational approaches
Source: Comput Struct Biotechnol J. 2023 Aug 29;21:4456–68. doi: 10.1016/j.csbj.2023.08.030 (PMC10511340; doi:10.1016/j.csbj.2023.08.030)
Supplement: Supplementary file 1 — Supplementary material. [file mmc1.pdf]

Supplementary figures

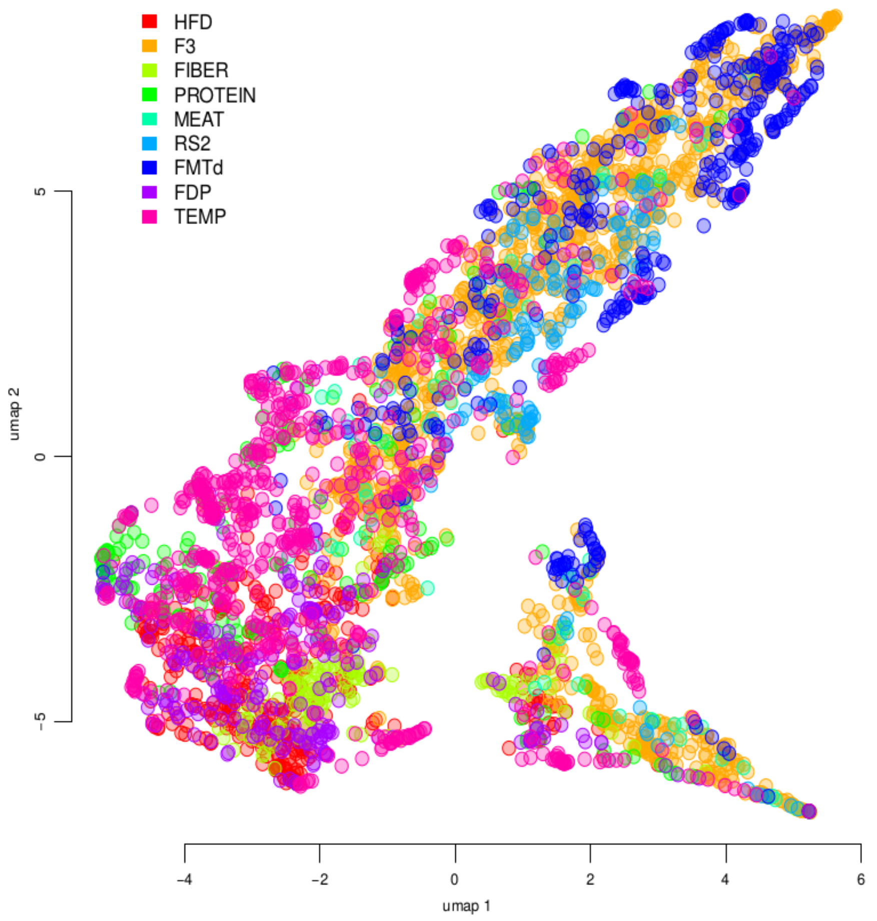

Supplementary Figure 1 - All samples included in the meta-analysis visualised using UMAP (N=3512).

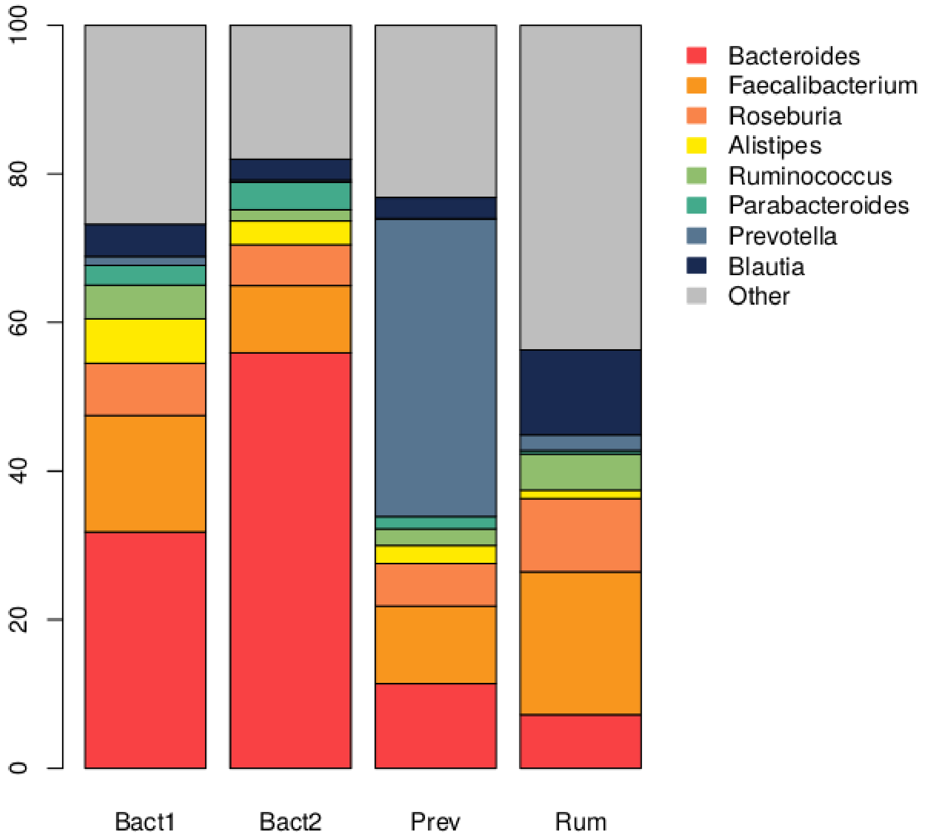

Supplementary Figure 2 - The most abundant taxa in the obtained enterotypes.

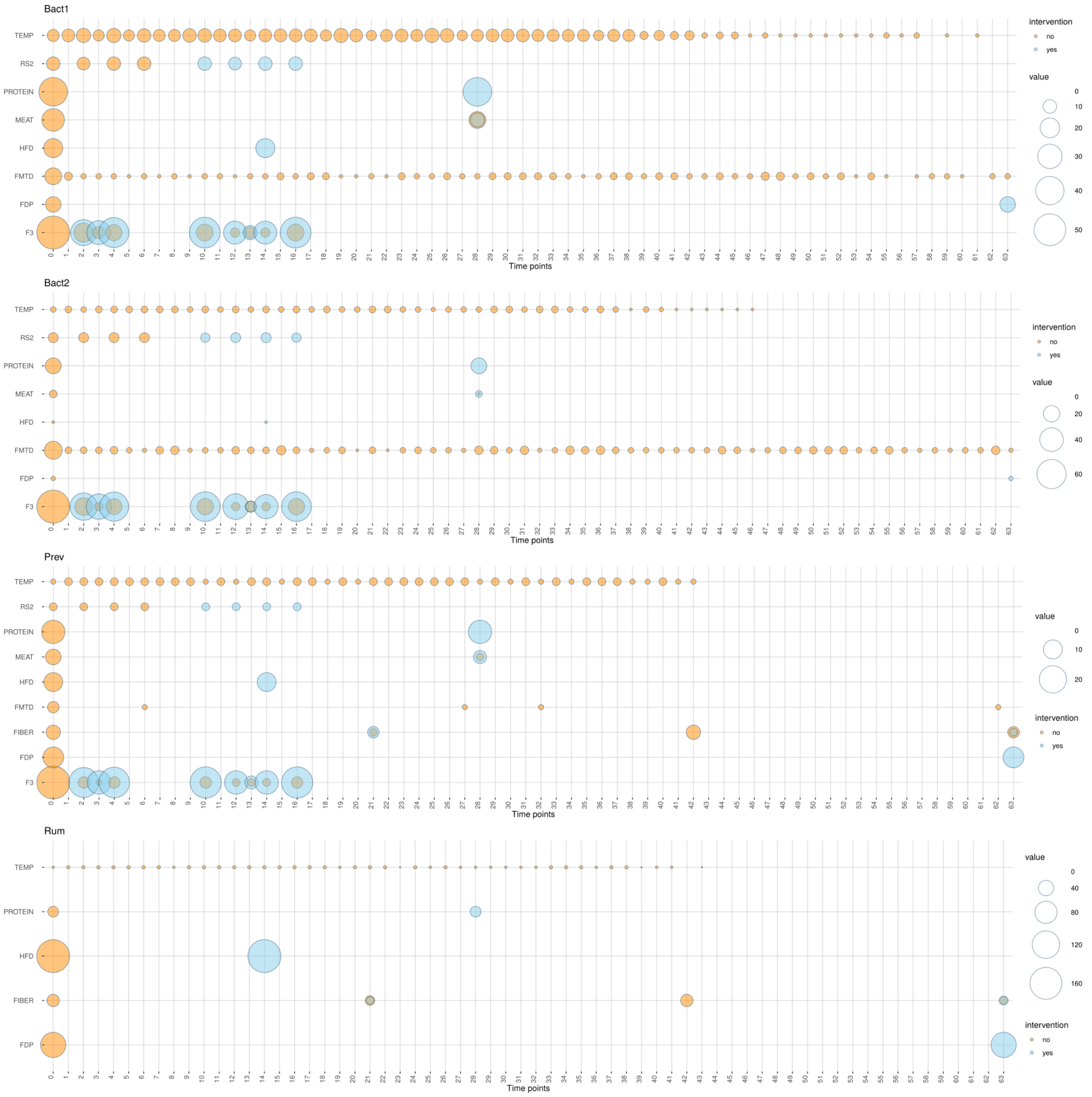

Supplementary Figure 3 - Distribution of the samples across time points and enterotypes for 9 studies. Color denotes whether a sample was collected after the intervention or not.

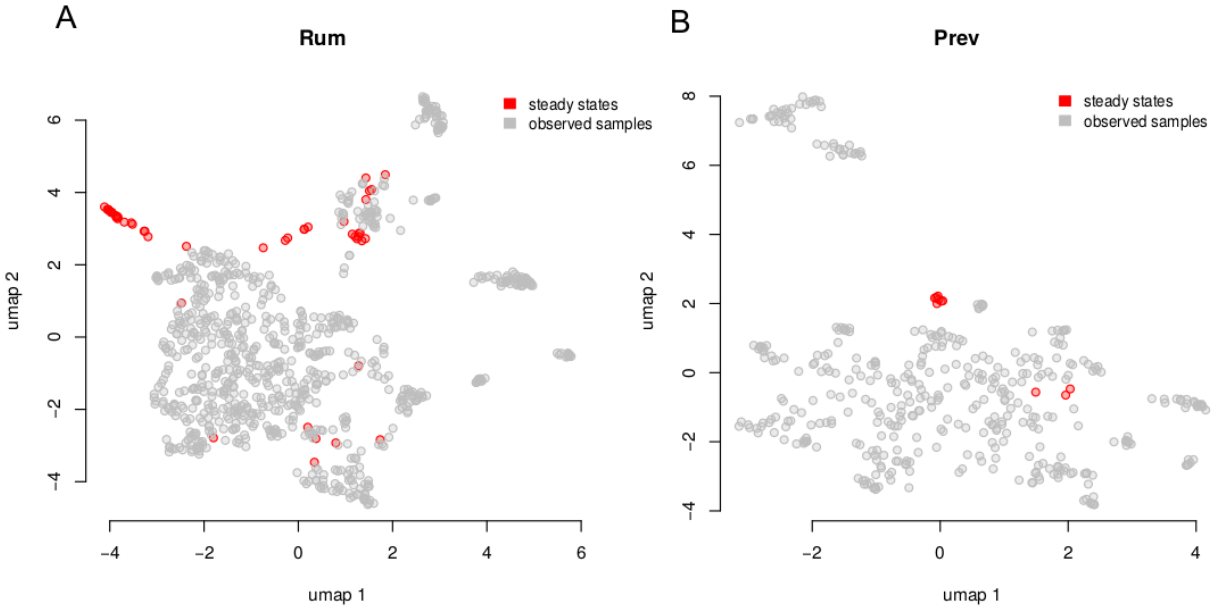

Supplementary Figure 4 - Samples from Rum (A) and Prev (B) enterotypes (Rum N=806, Prev N=411) and the steady states (Rum: N=51, Prev: N=10) obtained for the baseline samples using the trained cLV model; visualized using UMAP.

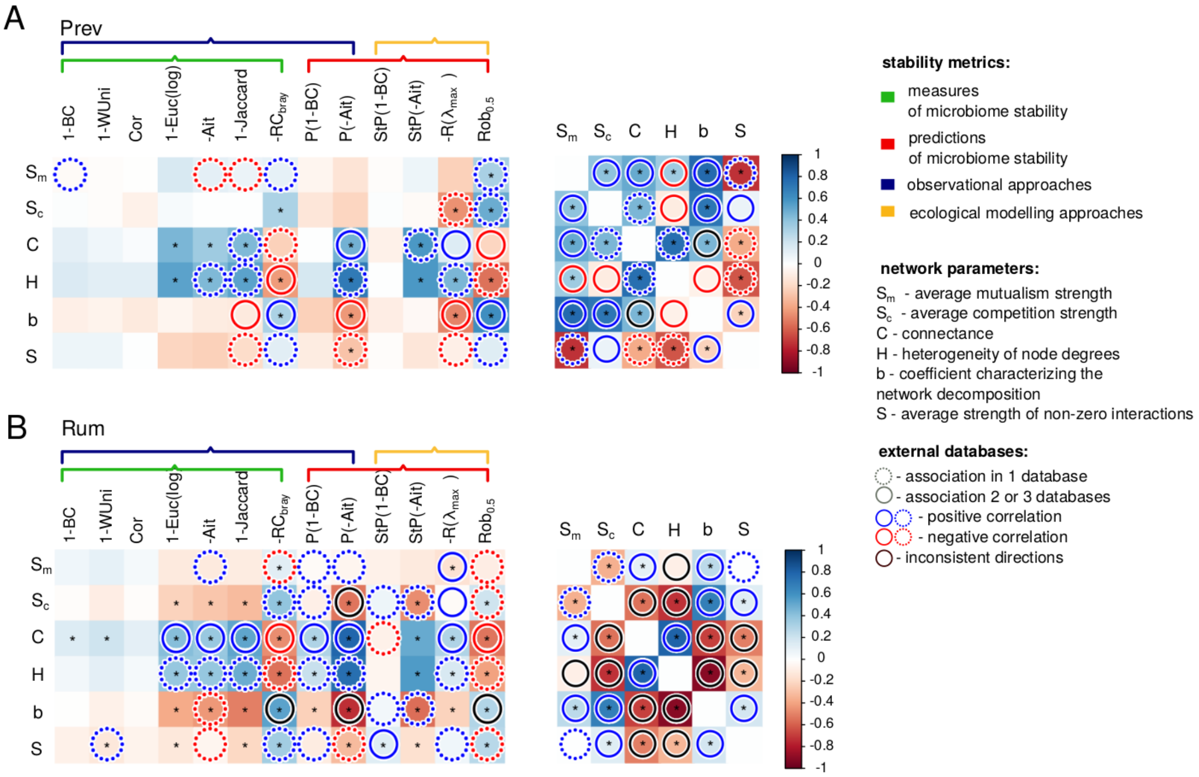

Supplementary Figure 5 - Correlations of relative interaction network properties - with stability estimates and within themselves. Stability metrics abbreviations are deciphered in the text. Colour denotes correlation coefficient. Asterisk denotes significant correlations (FDR<0.05). Circles denote significant associations detected when external databases were used for network parameters estimation (GutCP, MICOM, NJC19) (FDR<0.05). The number of samples for Prev/Rum enterotype is 88/325 respectively.
